# Initiation of once daily insulin detemir is not associated with weight gain in patients with type 2 diabetes mellitus: results from an observational study

**DOI:** 10.1186/1758-5996-5-56

**Published:** 2013-10-02

**Authors:** Jean-François Yale, Taner Damci, Marcel Kaiser, Eddy Karnieli, Kamlesh Khunti, Andreas Liebl, Florian MM Baeres, Anne Louise Svendsen, Stuart A Ross

**Affiliations:** 1 McGill Nutrition and Food Science Centre, Royal Victoria Hospital, Montreal, Quebec, Canada; 2 Department of Endocrinology, Cerrahpasa Medical School, Diabetes and Metabolism, Istanbul University, Istanbul, 34363, Turkey; 3 Practice for Internal Medicine and Diabetology, Frankfurt, Germany; 4 Endocrinology, Diabetes & Metabolism, Rambam Medical Center and Rappaport Faculty of Medicine, Technion, Haifa, Israel; 5 Diabetes Research Centre, University of Leicester, Leicester, UK; 6 Center for Diabetes and Metabolism, m&i-Fachklinik Bad Heilbrunn, Wörnerweg 30, Bad Heilbrunn, 83670, Germany; 7 Global Medical Affairs, Novo Nordisk A/S, Søborg, Denmark; 8 Department of Biostatistics & Epidemiology, Novo Nordisk A/S, Søborg, Denmark; 9 University of Calgary, Calgary, Alberta, Canada

**Keywords:** Type 2 diabetes mellitus, Insulin therapy, Weight, Body mass index, Insulin dose, Hypoglycaemia

## Abstract

**Background:**

Obesity is common in type 2 diabetes (T2DM) and is associated with increased risk of morbidity and all-cause mortality. This analysis describes weight changes associated with insulin detemir initiation in real-life clinical practice.

**Methods:**

Study of Once-Daily Levemir (SOLVE) was a 24-week international observational study of once-daily insulin detemir as add-on therapy in patients with T2DM receiving oral hypoglycaemic agents (OHAs).

**Results:**

17,374 participants were included in the analysis: mean age 62 ± 12 years; weight 80.8 ± 17.6 kg; body mass index (BMI) 29.2 ± 5.3 kg/m^2^; diabetes duration 10 ± 7 years; HbA1c 8.9 ± 1.6%. HbA1c decreased by 1.3 ± 1.5% during the study, with insulin doses of 0.27 ± 0.17 IU/kg. Patients with higher BMI had higher pre-insulin HbA1c, and similar reductions in HbA1c with insulin therapy. Weight decreased from 80.8 ± 17.6 kg to 80.3 ± 17.0 kg (change of -0.6 [95% CI -0.65; -0.47] kg), with 35% of patients losing >1 kg. Patients with the highest pre-insulin BMI lost the greatest amount of weight: BMI < 25: +0.8 [95% CI: 0.6; 0.9] kg, 25 ≤ BMI < 30: -0.2 [95% CI: -0.3; -0.8] kg, 30 ≤ BMI < 35: -1.0 [95% CI: -1.1; -0.8] kg; BMI ≥ 35: -1.9 [95% CI: -2.2; -1.6] kg. Minor hypoglycaemia decreased with increasing BMI: 2.3 and 1.3 events per patient year for BMI <25 and  ≥ 35, respectively.

**Conclusions:**

Overall, patients with poorly controlled T2DM achieved significant reductions in HbA1c after initiation of once-daily insulin detemir therapy, without weight gain. The favourable impact of insulin detemir on weight may not apply to other insulin preparations.

**Trial registrations:**

ClinicalTrials.gov, NCT00825643 and NCT00740519

## Introduction

The prevalence of type 2 diabetes mellitus (T2DM) and obesity is increasing worldwide. An estimated 80-90% of patients with T2DM are overweight, and obesity is acknowledged as a major factor in the development of T2DM [1]. Obesity in patients with T2DM is common and is frequently a major component of the metabolic syndrome, an insulin-resistant state that is characterized by a group of risk factors, which together confer an increased risk for cardiovascular disease and diabetes [2]. Insulin resistance is characterised by a reduced sensitivity in body tissues to the action of insulin, resulting in impaired glucose uptake in muscle and fat, and diminished insulin suppression of hepatic glucose output. As a result of insulin resistance, patients with T2DM require higher concentrations of insulin to stimulate peripheral glucose uptake and to suppress hepatic glucose production, than are needed in patients without diabetes [3].

It has become increasingly clear that physicians should give equal attention to weight management as they do to glycaemic control in patients with T2DM [4-8]. There is robust evidence to demonstrate that even a modest reduction in weight of between 5-10% in patients with T2DM has a beneficial effect in terms of improved glycaemic control, lowered blood pressure and an improvement in lipid profile [8,9]; and also lowers the risk of progression of diabetes, as well as cardiovascular disease [7]. Excessive weight is also an independent risk factor for increased morbidity and mortality in patients with T2DM [10].

Even though the early stages of T2DM may be managed with lifestyle changes and oral hypoglycaemic agents (OHAs), many patients with T2DM will eventually require insulin therapy. Typically, the initiation of insulin therapy has been associated with weight gain. Fear of weight gain by either patients and/or their health care providers is known to be one key reason why initiation of insulin is delayed despite being indicated on clinical grounds [11,12].

The objectives of this pre-specified sub-analysis from the Study of Once-Daily Levemir (SOLVE) study was to observe the impact of insulin detemir on weight and various cardio-metabolic parameters. To provide context, glycaemic control and hypoglycaemia are also briefly considered.

## Methods

SOLVE was a 24-week observational study in which insulin detemir was evaluated as add-on therapy in patients with T2DM already receiving OHAs. The study was conducted in ten countries (Canada, China, Germany, Israel, Italy, Poland, Portugal, Spain, Turkey and the UK). There were some variations between countries with respect to patient eligibility which have been reported previously [13]. The study was conducted in accordance with the Declaration of Helsinki. Ethical approval was obtained from local institutional review boards or independent ethics committees prior to the commencement of the study in each of the participating countries. The IRBs approving the study in the participating countries were: Health Canada (Canada), State Food and Drug Administration (China), Federal Institute for Drugs and Medicinal Devices (Germany), Ministry of Health (Israel), Agenzia Italiana del Farmaco (Italy), Ministry of Health (Poland), Comissão de Ética para a Saúde do Hospital do Divino Espírito Santo (Portugal), Spanish Agency for Medicines (Spain), Ministry of Health (Turkey), Medicines and Healthcare Products Regulatory Agency (UK).

### Patients

For all countries, the decision to initiate basal insulin preceded inclusion into the study and was made solely on the basis of a normal clinical evaluation as part of the patients’ routine diabetes management. Patients were recruited and followed up at primary and specialist health care facilities between February 2008 and January 2011.

Data were collected at baseline (including data prior to insulin initiation, where relevant), at 12 weeks (interim visit) and at 24 weeks (final visit), and were derived from patient recall, the patient’s clinical records and the patient’s self-monitored blood glucose (SMBG) diary.

The study excluded female patients who were pregnant, breast-feeding, intending to become pregnant within the next 6 months, or were not using adequate contraceptive methods. Patients had to be aged over 18 years to participate in four countries and over 6 years to participate in six countries. Withdrawal criteria included change in the once-daily dosing regimen of insulin detemir during the study, pregnancy, or the intention to become pregnant during the study. In these situations, the decision to discontinue insulin detemir was at the physician’s discretion. Patients could also withdraw from the study at any time and continue to receive normal clinical care.

### Endpoints

The primary outcome in SOLVE was the incidence of serious adverse drug reactions (SADRs) including severe hypoglycaemic events. Secondary efficacy endpoints included: glycosylated haemoglobin A1c (HbA1c), fasting blood glucose (FBG), self-monitored blood glucose (SMBG), weight and lipid profile. Physical measurements including blood pressure, weight, waist and hip circumference were recorded at baseline, interim and final visits. Information was also recorded about insulin therapy including dose and dose adjustments, and concomitant OHA therapy. Analysis of all major variables including change in body weight was performed for the total cohort and for various subgroups.

As the focus of this sub-analysis was the impact of insulin detemir initiation on weight and other cardio-metabolic parameters, the findings presented here focuses on four baseline body mass index (BMI) subgroups (BMI <25 kg/m^2^, 25 ≤ BMI <30 kg/m^2^, 30 ≤ BMI <35 kg/m^2^, BMI ≥35 kg/m^2^). These cut off points were pre-specified on the basis of being widely used in clinical trials of insulin and because of their use as internationally recognised definitions of overweight (BMI ≥25), obesity (BMI ≥30), and severe obesity (BMI ≥35) [14,15].

While detailed results are presented for these BMI subgroups, a summary of the major primary and secondary endpoints are also given for the entire cohort.

## Statistical methods

Two main analysis sets were used in this sub-analysis. The Full Analysis Set (FAS) comprised all enrolled patients who had been prescribed basal insulin detemir at baseline. The Effectiveness Analysis Set (EAS) comprised all patients from the FAS with a final visit between 16 and 32 weeks, and at least one FBG, HbA1c, weight measurement or record of hypoglycaemia at both baseline and final visit. The EAS was used for the analyses of HbA1c, blood glucose and lipid profiles. The FAS was used for the reporting of baseline characteristics, and analysis of all other variables (including ADRs and hypoglycaemia).

Continuous variables were summarized using mean and standard deviation, and categorical data were summarized as counts (percentages). Missing observations were not included in the calculation of percentages.

Statistical testing of data before and after treatment with insulin detemir was analysed using paired t-tests for continuous variables, the Wilcoxon test for ordinal categorical variables, and the McNemar test for discrete variables such as incidence of hypoglycaemic events.

The influence of predictor variables on the reduction in weight ≥1 kg was evaluated by logistic regression analysis. The basic model included age (in 5-year groupings), gender, duration of diabetes (in quartiles), pre-insulin HbA1c (as a continuous variable) and BMI subgroup. The effects of previous medical history and number of OHAs were assessed for the basic model parameters. Additional assessment of the effects of individual OHAs during treatment with insulin, change in OHA prescribing at the time of insulin initiation and insulin dose (mean and final insulin dose) were adjusted for basic model parameters and number of OHAs prescribed pre-insulin. All parameters were included in the final backward elimination model, with the criteria for selection set at p < 0.05. All testing used two-sided tests at α = 0.05 level of significance.

## Results

A total of 17,374 participants were included in the FAS. A total of 2,761 patients withdrew from the study, of which 1,548 (n = 1548/17372, 8.9%) patients did so at or before the interim visit and 1,213 (n = 1213/17372, 7.0%) discontinued at or before the final visit. Reasons for study withdrawal included lost to follow-up (34%; n = 934), discontinuation of OHA (5%; n = 144), discontinuation of study drug (11%; n = 297), incorrect study drug regimen (9%; n = 237), addition of short acting insulin (15%; n = 417), ADR (3%; n = 87), and other reasons (21%; n = 588). The reason for study withdrawal was missing for 13% of patients (n = 363). Patients could report more than one reason for study withdrawal.

### Patient demography

Baseline characteristics for the total cohort as well as for BMI subgroups are summarised in Table 1. The mean weight (±SD) at baseline was 80.8 ± 17.6 kg for the overall cohort and the mean BMI was 29.2 ± 5.3 kg/m^2^. Patients with a BMI ≥35 kg/m^2^ at baseline tended to be younger, and there were a higher proportion of female patients relative to the other BMI subgroups.

**Table 1:** Baseline characteristics

	**Total cohort**	**Body mass index (BMI) at baseline**			
		**BMI <25**	**25 ≤ BMI < 30**	**30 ≤ BMI < 35**	**BMI ≥ 35**
N (%)	17,374	3,534 (20.8%)	6,811 (40.1%)	4,218 (24.9%)	2,405 (14.2%)
Age (years)	62 ± 12	62 ± 12	62 ± 12	62 ± 11	60 ± 11
Male (%)	53%	55%	58%	52%	39%
Weight (kg)	80.8 ± 17.6	62.8 ± 8.5	76.4 ± 9.2	88.6 ± 11.0	104.5 ± 15.6
BMI (kg/m^2^)	29.2 ± 5.3	22.8 ± 1.7	27.4 ± 1.4	32.1 ± 1.4	38.8 ± 3.3
Waist circumference (cm)	99.3 ± 15.1	85.3 ± 9.0	96.0 ± 9.7	107.3 ± 9.4	119.5 ± 12.4
Hip circumference (cm)	104.0 ± 13.4	94.5 ± 8.0	102.3 ± 8.4	110.7 ± 9.7	123.3 ± 14.0
Waist:hip ratio	0.94 ± 0.11	0.90 ± 0.09	0.93 ± 0.08	0.97 ± 0.10	0.98 ± 0.13
Blood pressure (mmHg)					
Systolic	136 ± 17	131 ± 16	135 ± 16	139 ± 16	140 ± 18
Diastolic	81 ± 10	78 ± 9	81 ± 9	82 ± 10	82 ± 11
FBG (mmol/L)	10.3 ± 3.1	9.8 ± 3.0	10.0 ± 2.9	10.4 ± 3.1	10.8 ± 3.1
HbA1c (%)	8.9 ± 1.6	8.7 ± 1.8	8.8 ± 1.5	9.0 ± 1.5	9.1 ± 1.5
Lipd profile (mmol/L)					
Total cholesterol	5.1 ± 1.2	5.0 ± 1.1	5.1 ± 1.2	5.1 ± 1.2	5.0 ± 1.2
HDL	1.2 ± 0.4	1.3 ± 0.4	1.2 ± 0.3	1.2 ± 0.4	1.2 ± 0.3
LDL	2.9 ± 1.0	2.9 ± 0.9	3.0 ± 1.0	2.9 ± 1.0	2.8 ± 1.0
Triglycerides	2.1 ± 1.2	1.8 ± 1.2	2.1 ± 1.2	2.2 ± 1.2	2.4 ± 1.2
Duration of Diabetes (years)	10 ± 7	10 ± 8	10 ± 7	10 ± 7	9 ± 7
Duration of OHA treatment	8 ± 7	9 ± 8	8 ± 6	8 ± 6	8 ± 7
Number of OHAs					
1 OHA	29.9%	33.7%	30.3%	26.8%	26.9%
2 OHAs	54.1%	50.4%	54.8%	56.7%	54.2%
>2 OHAs	16.0%	15.8%	14.9%	16.5%	18.9%

Continuous measures are shown as mean ± standard deviation.

Glycaemic control (as measured by HbA1c and FBG) deteriorated in association with increasing baseline BMI. HbA1c was 9.1 ± 1.5% in patients with a BMI ≥35 kg/m^2^ compared with 8.7 ± 1.8% in patients with a BMI <25 kg/m^2^.

Baseline blood pressure and triglycerides also increased in association with increasing BMI. Mean systolic blood pressure at baseline was 131 ± 16 mmHg in patients with a BMI <25 kg/m^2^ and 140 ± 18 mmHg in patients with a BMI ≥35 kg/m^2^. Mean triglycerides levels at baseline were 2.4 ± 1.2 mmol/L in patients with a BMI ≥35 kg/m^2^ compared with 1.8 ± 1.2 mmol/L in patients with a BMI <25 kg/m^2^.

### Glycaemic efficacy

Glycaemic control improved in each of the BMI subgroups, with statistically significant reductions in HbA1c and FBG at the final visit compared with pre-insulin values in all subgroups (Table 2).

**Table 2:** Glycaemic control, hypoglycaemia, weight, size, lipid profile and blood pressure by BMI subgroup

	**BMI <25**	**25 ≤ BMI < 30**	**30 ≤ BMI < 35**	**BMI ≥ 35**
	**(n = 3534)**	**(n = 6811)**	**(n = 4218)**	**(n = 2405)**
**Glycaemic control**				
HbA1c (%)				
Final visit	7.4 ± 1.2	7.5 ± 1.1	7.7 ± 1.1	7.9 ± 1.3
Change	-1.4 ± 1.7**	-1.3 ± 1.5**	-1.3 ± 1.5**	-1.3 ±1.5**
FBG (mmol/L)				
Final visit	6.8 ± 1.7	7.0 ± 1.6	7.2 ± 1.8	7.7 ± 2.2
Change	-3.0 ± 3.0**	-3.0 ± 2.9**	-3.2 ± 2.9**	-3.2 ± 3.1**
**Hypoglycaemia Incidence (events per patient year)**				
Severe hypoglycaemia				
Pre-insulin	0.057	0.048	0.036	0.028
Final visit	0.004	0.004	0.010	0.004
Change	-0.053**	-0.044**	-0.026**	-0.024
Minor hypoglycaemia				
Pre-insulin	1.755	1.583	1.341	1.699
Final visit	2.390	2.009	1.471	1.292
Change	+0.635**	+0.426*	+0.130*	-0.407
**Weight (kg)**				
Change	+0.75 [0.60; 0.90]	**-**0.16 [-0.26; -0.07]	-0.99 [-1.13; -0.84]	-1.89 [-2.16; -1.63]
**Change in Anthropometric measurements (cm)**				
Waist circumference	0.10 [-0.12; 0.32]	-0.51 [-0.72; -0.29]	-1.23 [-1.57; -0.88]	-2.18 [-2.64; -1.73]
Hip circumference	-0.32 [-0.56;- 0.07]	-0.67 [-0.88; -0.47]	-0.86 [-1.35; -0.36]	-1.30 [-1.90; -0.70]
**Change in lipid profile (mmol/L)**				
Total cholesterol	-0.26 [-0.31; -0.20]	-0.31 [-0.35; -0.26]	-0.34 [-0.40; -0.27]	-0.35 [-0.42; -0.27]
HDH cholesterol	+0.01 [-0.01; 0.03]	+0.01 [0.00; 0.02]	+0.01 [-0.01; 0.03]	+0.02 [-0.01; 0.04]
LDL cholesterol	-0.15 [-0.19; -0.10]	-0.16 [-0.20; -0.12]	-0.18 [-0.24; -0.12]	-0.20 [-0.28; -0.13]
Triglycerides	-0.23 [-0.29; -0.17]	-0.26 [-0.31; -0.22]	-0.34 [-0.40; -0.28]	-0.44 [-0.52; -0.37]
**Change in blood pressure (mmHg)**				
Systolic	-3.03 [-3.59; -2.46]	-3.77 [-4.23; -3.31]	-5.06 [-5.70; -4.43]	-5.10 [-5.97; -4.24]
Diastolic	-1.26 [-1.62; -0.91]	-2.31 [-2.59; -2.03]	-2.22 [-2.60; -1.84]	-2.24 [-2.77; -1.71]

Continuous measures are shown as mean ± standard deviation or mean [95% confidence interval].

**p < 0.001.

*p < 0.01.

**Figure 1:**
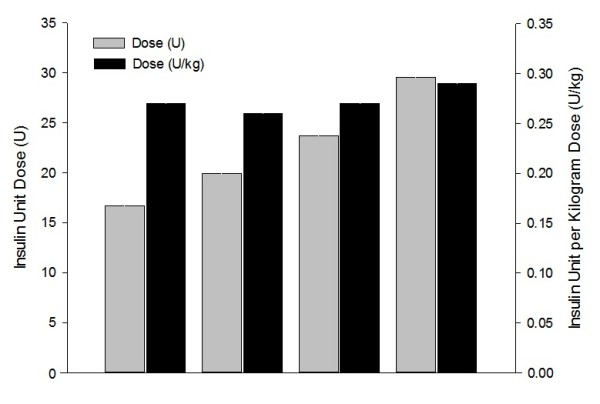
Insulin dose in units and units per kilogram at final visit by BMI subgroup.

The insulin dose according to BMI subgroups are shown in Figure 1. The figure demonstrates the contrast between the trend to increasing unit dose but similar unit per kilogram dose with with increasing BMI.

### Hypoglycaemia

There were statistically significant reductions in the rates of severe hypoglycaemia in all BMI subgroups (Table 2). Whilst there was an increase in the incidence of minor hypoglycaemia in three of the four BMI subgroups, there was a reduction in the rate of minor hypoglycaemia in the subgroup of patients with the largest BMI (-0.41 events per person year), although this did not reach statistical significance (p = 0.116).

### Weight

Small reductions in weight, BMI, and waist and hip circumference were observed across the total cohort following the initiation of insulin detemir. Mean weight changed by -0.56 kg, [95% CI -0.65; -0.47] from a pre-insulin value of 80.8 ± 17.6 kg to a final visit value of 80.3 ± 17.0 kg (p < 0.001). Similarly, mean BMI changed by -0.2 kg/m^2^ from a pre-insulin value of 29.2 ± 5.3 kg/m^2^ to a final visit value of 29.0 ± 5.2 kg/m^2^ (p < 0.001). Mean waist circumference changed by -0.69 cm (from 98.3 ± 14.8 cm to 97.6 ± 14.4 cm; p < 0.001) and mean hip circumference changed by -0.66 cm (from 103.5 ± 12.9 cm to 102.8 ± 12.6 cm; p < 0.001).

The degree of weight change was proportional to the pre-insulin BMI (Table 2): patients with the highest pre-insulin BMI ≥35 kg/m^2^ lost the greatest amount of weight (-1.9 kg; p < 0.001), while patients with pre-insulin BMI <25 kg/m^2^ had a weight gain (+0.75 kg; p < 0.001).

The proportion of patients who lost more than 1kg during the study increased in association with larger pre-insulin BMI: (49% of patients with BMI ≥35 kg/m^2^ compared with 42%, 32% and 19% of patients with pre-insulin BMI of: 30 ≤ BMI <35 kg/m^2^, 25 ≤ BMI <30 kg/m^2^, and <25 kg/m^2^, respectively).

### Blood pressure and lipids

Treatment with insulin detemir was associated with improvements in patients’ lipid profiles. At the final visit, there were significant reductions (p < 0.001) in total cholesterol (-0.30 mmol/L), low-density lipoprotein (LDL) cholesterol (-0.16 mmol/L) and triglycerides (-0.29 mmol/L) across the entire cohort. Significant improvements in lipid profile also occurred in all BMI subgroups, with a tendency towards greater improvements in subgroups with a higher BMI (Table 2), particularly for triglycerides.

There was a significant decrease in systolic and diastolic blood pressure across the entire cohort at the final visit compared with pre-insulin values (systolic: mean change -4 mmHg, p < 0.001; diastolic: mean change -2 mmHg, p < 0.001). While blood pressure tended to increase with baseline BMI, reductions in blood pressure during the study were also greater in patients with larger BMI (Table 2).

### OHA use

Some differences in OHA therapy prior to insulin initiation were apparent between BMI subgroups (Table 3). For example, there was a trend towards increasing pre-insulin use of biguanides, thiazolidinediones and dipeptidyl peptidase IV (DPP-IV) inhibitors in patients with larger BMI; whereas there was a trend in the opposite direction concerning the pre-insulin use of glinides and α-glucosidase inhibitors. With the exception of glinide and α-glucosidase inhibitor use, the proportion of patients using each of the classes of OHA decreased from pre-insulin to the end of the study, with the greatest percentage reductions occurring in patients with BMI ≥35 kg/m^2^ (Table 3). The largest change in the use of OHA during the study was with respect to sulphonylurea, which decreased by 15.0% in the subgroup with BMI ≥35 kg/m^2^ and by 16.5% in the subgroup with BMI <25 kg/m^2^. The proportion of patients using glinides during the study, however, increased in all BMI subgroups.

**Table 3:** Proportion of patients using oral antidiabetic drugs (OHA) prior to insulin initiation and at end of study

	**Body mass index (BMI) at baseline**			
	**BMI <25**	**25 ≤ BMI < 30**	**30 ≤ BMI < 35**	**BMI ≥ 35**
**Biguanide**				
Baseline	71.2%	81.8%	85.8%	87.0%
Final Visit	65.1%	80.5%	85.2%	86.5%
Change	-6.1%	-1.3%	-0.6%	-0.5%
**Glinides**				
Baseline	19.5%	16.3%	14.7%	13.5%
Final Visit	24.7%	18.8%	17.1%	16.1%
Change	+5.2%	+2.5%	+2.4%	+2.6%
**Alpha Glucosidase Inhibitor**				
Baseline	19.9%	12.2%	8.6%	7.8%
Final Visit	21.4%	12.5%	7.8%	6.6%
Change	+1.5%	+0.3%	-0.8%	-1.2%
**Sulphonylureas**				
Baseline	57.9%	58.5%	61.7%	60.3%
Final Visit	41.4%	43.3%	46.4%	45.3%
Change	-16.5%	-15.2%	-15.3%	-15.0%
**Thiazolidinediones**				
Baseline	10.9%	11.3%	12.8%	15.2%
Final Visit	7.3%	7.9%	7.7%	8.2%
Change	-3.6%	-3.4%	-5.1%	-7.0%
**Dipeptidyl peptidase IV Inhibitors**				
Baseline	3.8%	5.9%	7.9%	9.6%
Final Visit	2.8%	5.2%	6.5%	7.2%
Change	-1.0%	-0.7%	-1.4%	-2.4%

### Predictors of weight gain

Logistic regression analysis was performed in order to evaluate demographic and treatment parameters associated with weight loss of 1 kg or more. Results of the basic model, and basic model adjusted effects are shown in Table 4. In the basic model, lower pre-insulin HbA1c and increased pre-insulin BMI were associated with a significantly increased odds ratio (OR) of weight loss  ≥ 1 kg. Adjusting for basic model parameters, the OR of weight loss ≥ 1 kg decreased with increasing number of pre-insulin OHAs. After additionally adjusting for the number of pre-insulin OHAs, higher doses of insulin and the concomitant use of thiazolidinediones were also associated with significantly lower odds of weight loss ≥ 1 kg; whereas a reduction in the number of concomitant OHAs prescribed or the use of biguanides at the time of insulin initiation was associated with an increase in the odds of weight loss ≥ 1 kg.

**Table 4:** Results of logistic regression models of demographic and study treatment parameters associated with weight loss ≥ 1 kg by final visit

**Parameter**	**Category**	**Odds ratio**	**95% confidence interval**		**p value**
			**Lower**	**Upper**	
**Basic Model**					
Baseline HbA1c (%)		0.927	0.905	0.950	<0.0001
Age (years)	≥75	0.902	0.776	1.049	0.6660
	≥70 to < 75	0.969	0.834	1.126	
	≥65 to < 70	0.959	0.829	1.109	
	≥60 to < 65	1.015	0.885	1.165	
	≥55 to < 60	1.020	0.889	1.171	
	≥50 to < 55	0.945	0.813	1.098	
	< 50 (reference group)	0	.	.	
Gender	Female vs. Male	0.916	0.849	0.989	0.0229
Duration of Diabetes (years)	>13	0.945	0.844	1.059	0.1681
	>8.5 to ≤13	0.884	0.795	0.983	
	>5 to ≤8.5	0.898	0.807	1.000	
	≤5 (reference group)	0	.	.	
BMI (kg/m^2^)	≥35	3.351	2.934	3.827	<0.0001
	≥30 to <35	2.389	2.130	2.681	
	≥25 to <30	1.709	1.538	1.899	
	<25 (reference group)	0	.	.	
**Adjusted for Basic Model Parameters**					
Previous History					
Pre-insulin Hypoglycaemia	Yes vs. No	1.113	0.940	1.318	0.4518
Microvascular disease	Yes vs. No	1.050	0.967	1.141	0.4390
Macrovascular disease	Yes vs. No	1.021	0.934	1.116	0.4052
Number of OHAs Prior to Insulin Initiation	>2 OHAs	0.853	0.756	0.963	0.0003
	2 OHAs	0.928	0.850	1.012	
	1 OHA (reference group)	0	.	.	
**Adjusted for Basic Model Parameters and Number of OHAs Prior to Insulin Initiation**					
Change in OHA Regimen	Increased	0.965	0.784	1.189	0.0002
	Decreased	1.150	1.035	1.278	
	Unchanged (reference group)	0	.		
Concomitant OHA Treatment					
Metformin	Yes vs. No	1.167	1.066	1.279	0.0009
Sulphonylureas	Yes vs. No	0.962	0.888	1.043	0.3485
Glinides	Yes vs. No	0.939	0.848	1.041	0.2315
α-Glucosidase Inhibitors	Yes vs. No	1.102	0.973	1.248	0.1262
Thiazolidinediones	Yes vs. No	0.789	0.675	0.921	0.0027
DPP-IV Inhibitors	Yes vs. No	0.845	0.710	1.005	0.0576
Final Insulin Dose (U/kg)	>0.3	0.871	0.773	0.981	0.0241
	0.2 to <0.3	0.887	0.791	0.994	
	0.1 to <0.2	0.973	0.877	1.079	
	< 0.1 (reference group)	0	.	.	
Mean Insulin Dose (U/kg)	>0.27	0.836	0.740	0.945	0.0193
	0.20- < 0.27	0.880	0.776	0.997	
	0.13- < 0.20	0.944	0.850	1.049	
	<0.13 (reference group)	0			

The effect size is expressed as Odds Ratios (OR) with 95% confidence intervals (CI).

**Figure 2:**
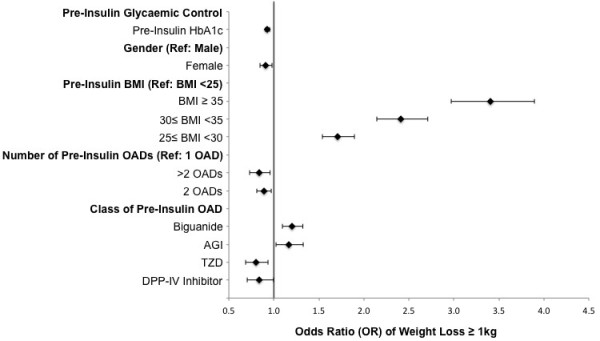
**Demographic and treatment parameters associated with weight loss ≥ 1 kg by final visit – results of a final backward elimination logistic regression model.** Error bars indicate 95% confidence intervals.

In the final backward elimination model, pre-insulin HbA1c, gender, BMI, the number of OHAs, and the concomitant use of metformin (positive), α-glucosidase inhibitor (positive), thiazolidinedione (negative) and DPP-IV inhibitor (negative) all had significant independent effects on the odds of weight loss ≥ 1 kg; with the largest effect determined by pre-insulin BMI (Figure 2). In this final model, insulin dose (mean insulin dose or final insulin dose as measured in U/kg) was not independently associated with weight loss ≥ 1 kg.

## Discussion

This large observational study of patients with T2DM demonstrated that the initiation of once daily insulin detemir in patients already receiving OHA therapy was effective in improving glycaemic control, and was associated with a low incidence of hypoglycaemia and an overall mean reduction in weight across the entire cohort. Mean weight reductions occurred in all patient subgroups with a BMI ≥25 kg/m^2^. This weight-sparing effect was most apparent in overweight and obese patients, with changes in weight of up to -1.9 kg in the subgroup of patients with BMI ≥35 kg/m^2^. Treatment with insulin detemir was also associated with improvements in other cardio-metabolic parameters including lipid profile and a reduction in blood pressure.

Although baseline HbA1c was higher in patients with larger BMI, the reduction in HbA1c was comparable across all BMI subgroups (-1.3 to -1.4%). Conventional understanding would suggest that increased insulin resistance in patients with higher BMI necessitates higher insulin per kilogram dosing. In the present study, the unit dose of insulin increased with increasing BMI, however, the unit per kilogram dose was similar across all four BMI subgroups (0.26-0.29 units/kg at the final visit). The fact that the majority of patients did not reach the usual HbA1c target of 7.0% in the absence of any increase in hypoglycemic episodes suggests a degree of clinical inertia in adjusting insulin dosages. This is supported by the lower insulin dosages in the present study compared to those reported in treat-to-target trials (0.4 and 0.6 U/kg) [16-19]. It is possible that the effects on body weight would have been different with a more aggressive insulin initiation regimen, or over longer periods of follow-up.

Recent reviews examining the effect of OHA treatment either alone or in combination with insulin have shown that, in general, the sulphonylureas and thiazolidinediones promote weight gain, DPP-IV inhibitors are weight neutral, while biguanides (Metformin) promote weight loss [5,20,21]. In the present analysis, the logistic regression model showed that both biguanides (increased odds) and thiazolidinediones (decreased odds) were independent predictors of weight loss ≥1 kg at final visit. However, a greater proportion of patients with larger BMI were prescribed biguanides and thiazolidinediones. Furthermore, the use of thiazolidinediones decreased across all BMI subgroups (to a greater extent in patients with larger BMI). In this study, the type, frequency and dosing of OHAs could be modified at the discretion of the physician. The pattern of OHA treatment observed in this study may also have been related to country-specific usage of these medications. It is possible that differences in OHA therapy observed prior to and during the study, may have contributed to weight change differences between the BMI groups; however, it is unlikely, given the effect size of BMI subgroup on the odds of weight loss ≥1 kg (Table 4), that BMI related effects on weight loss during treatment with insulin detemir can be explained by the differences in OHA therapy alone.

Basal insulin analogues such as insulin detemir were developed using recombinant techniques to provide a more physiological profile than human intermediate insulin preparations, and have been shown to be effective and safe in several randomized controlled trials, with a reduced risk of hypoglycaemia compared with traditional human insulins [22]. Additionally, insulin detemir is associated with a unique overall weight-sparing effect in patients with diabetes. The results from SOLVE add to a growing body of data, including randomised controlled trials pooled analyses, and other real-world data demonstrating a weight-sparing effect of insulin detemir [23-26]. The weight-sparing effect appears to be greatest in the most overweight patients [24,26]. This relationship is of considerable clinical significance since it is the heaviest group of patients that would also be expected to be at the greatest risk of developing macrovascular (and in particular, cardiovascular) complications. By contrast, other long acting insulin analogues, such as insulin glargine, do not appear to share this weight–sparing effect – an analysis of 4555 patients with BMI < and ≥ 30 demonstrated a mean weight increase in both groups (+1.2 kg and +1.1 kg, respectively) [27].

The mechanisms for the weight-sparing effect of insulin detemir, independent of changes in concomitant OHA therapy, remain to be elucidated, but various possibilities have been suggested [28,29]. One explanation is that the reduced risk of hypoglycaemia with insulin detemir compared with traditional human insulin products may reduce the need for defensive snacking (over eating in order to try to prevent hypoglycaemic episodes). However, insulin glargine, which reduces hypoglycaemia to a similar degree, does not appear to have this weight-sparing effect. A second explanation relates to its novel method of prolonging action via acylation and albumin binding, resulting in preferential hepatic utilisation, thus suppressing hepatic glucose output without promoting lipogenesis in the periphery. A third hypothesis is that insulin detemir may be more effective than traditional human insulin in signalling satiety within the central nervous system [30]. Further research of the mechanisms underlying insulin detemir’s weight-sparing properties is required.

Given the observational nature of this study, definitive conclusions about the extent to which insulin detemir was responsible for all the measured changes are limited. Only patients initiating basal insulin therapy with insulin detemir were included in the analyses. This may mean, for example, that the cohort is not representative of patients requiring additional prandial glucose coverage at the time of insulin initiation. Nevertheless, SOLVE is the largest study of insulin initiation in real-life clinical practice, and otherwise broad inclusion criteria and the focus on insulin initiation in type 2 diabetes are important study strengths.

## Conclusions

The SOLVE study adds to the growing body of evidence showing that insulin detemir is associated with an overall weight-sparing effect which has not been demonstrated by other insulin preparations. This weight-sparing property appears to be most pronounced in the heaviest patients, a group that stands to gain the most benefit from losing weight. The management of weight is essential at all stages of treatment of patients with T2DM and should be given equal importance to achieving glycaemic control owing to the association with risk of developing cardiovascular complications.

## Competing interests

All authors have received consulting fees and support for travel to meetings from Novo Nordisk in association with the SOLVE study.

JFY has consulted for or received lecture fees from Astra Zeneca, Boehringer-Ingelheim, Bristol Meyers Squibb, Eli Lilly, Medtronic, Merck, Novartis, Novo Nordisk and Sanofi-Aventis; and his institution has received grants from Astra Zeneca, Bristol Meyers Squibb, Eli Lilly, Medtronic, Merck, Novartis, Novo Nordisk and Sanofi-Aventis. TD has received consulting fees from Novo Nordisk, Sanofi-Aventis, Eli Lilly, Merck, Bristol Meyers Squibb, and Astra Zeneca. EK has received consulting and lecture fees from Novo Nordisk. MK has consulted for Novo Nordisk. KK has received consulting and lectures fees from Novo Nordisk, Eli Lilly, Merck, Bristol Meyers Squibb, Roche and Boeringer; his institution has received a grant from NIHR CLAHRC to study depression and diabetes. AL has received consulting and/or lecture fees from Novo Nordisk, Merck, Roche, and Astra Zeneca. SR has received honoraria and research grants from Eli Lilly, Novo Nordisk and Sanofi Aventis. ALS and FMMB are employees of Novo Nordisk.

## Authors’ contributions

JFY and KK gave input to the study protocol and pre-defined subanalysis of the effect of baseline BMI. JFY, TD, EK, MK, KK, AL and SR have all been involved in the day to day running, interpretation and reporting of the study. ALS performed and/or reviewed all statistical analyses. The manuscript outline was prepared with JFY. All authors have given subsequent input to the drafts of this manuscript, and have reviewed and approved all content.

## Authors’ information

JFY and KK are members of the SOLVE study steering committee responsible for protocol development and overseeing the conduct of the study. TD, MK, EK, AL and SR are principle investigators for their respective countries.
